# Neuropsychiatric Adverse Drug Reactions with Tyrosine Kinase Inhibitors in Gastrointestinal Stromal Tumors: An Analysis from the European Spontaneous Adverse Event Reporting System

**DOI:** 10.3390/cancers15061851

**Published:** 2023-03-20

**Authors:** Maria Antonietta Barbieri, Emanuela Elisa Sorbara, Giulia Russo, Giuseppe Cicala, Tindara Franchina, Mariacarmela Santarpia, Nicola Silvestris, Edoardo Spina

**Affiliations:** 1 Department of Clinical and Experimental Medicine, University of Messina, 98125 Messina, Italy; 2 Department of Human Pathology in Adulthood and Childhood Gaetano Barresi, University of Messina, 98125 Messina, Italy

**Keywords:** tyrosine kinase inhibitors (TKI), gastrointestinal stromal tumors, neuropsychiatric disorders, adverse drug reactions, safety monitoring

## Abstract

**Simple Summary:**

Gastrointestinal stromal tumors (GISTs) are rare mesenchymal neoplasms arising in the gastrointestinal tract, especially in the stomach (60–65%). The development of most cases of GISTs is associated with activating mutations in the receptor tyrosine kinase genes KIT and PDGFRA. The discovery of mutations leads to the approval of tyrosine kinase inhibitors (TKIs) for GISTs. Considering the recent approval of the last two TKIs and given their increased use in clinical practice, a spontaneous reporting system (SRS) database, the main tool in pharmacovigilance, takes a remarkable role in promptly characterizing the benefit/risk profile of these new drugs and consequently improve the quality of life of patients affected by GISTs.

**Abstract:**

Tyrosine kinase inhibitors (TKIs) are widely used in gastrointestinal stromal tumors (GISTs). The aim of this study is to evaluate the reporting frequency of neuropsychiatric adverse drug reactions (ADRs) for TKIs through the analysis of European individual case safety reports (ICSRs). All ICSRs collected in EudraVigilance up to 31 December 2021 with one TKI having GISTs as an indication (imatinib (IM), sunitinib (SU), avapritinib (AVA), regorafenib (REG), and ripretinib (RIP)) were included. A disproportionality analysis was performed to assess the frequency of reporting for each TKI compared to all other TKIs. The number of analyzed ICSRs was 8512, of which 57.9% were related to IM. Neuropsychiatric ADRs were reported at least once in 1511 ICSRs (17.8%). A higher reporting probability of neuropsychiatric ADRs was shown for AVA. Most neuropsychiatric ADRs were known, except for a higher frequency of lumbar spinal cord and nerve root disorders (reporting odds ratio, ROR 4.46; confidence interval, CI 95% 1.58–12.54), olfactory nerve disorders (8.02; 2.44–26.33), and hallucinations (22.96; 8.45–62.36) for AVA. The analyses of European ICSRs largely confirmed the safety profiles of TKIs in GISTs, but some ADRs are worthy of discussion. Further studies are needed to increase the knowledge of the neuropsychiatric disorders of newly approved TKIs.

## 1. Introduction

Gastrointestinal stromal tumors (GISTs) are rare mesenchymal neoplasms arising in the gastrointestinal tract, especially in the stomach (60–65%), representing less than 1% of all digestive tumors, and are characterized by variable malignant potential and clinical behavior [1,2]. The annual incidence of GISTs is estimated to have a variable range from 0.4 to 2 cases per 100,000 people [2]. GISTs arise from the interstitial cells of Cajal, which are pacemakers of the gastrointestinal tract responsible for intestinal contractions [3]. Moreover, the development of most cases of GISTs is associated with activating mutations in the receptor tyrosine kinase genes KIT and PDGFRA: KIT primary mutations are found in 75–80% of GISTs, mainly involving exon 11 or 9 while only rare cases affect exon 13 or 17 [4], and 10–20% of GISTs contain oncogenic mutations of PDGFRA, particularly in exons 18, 12, and 14. The D842V mutation, based on the substitution of aspartic acid to valine, is the most common exon 18 mutation. In KIT/PDGFRA wild-type GISTs, other oncogenic drivers have been identified, including mutations of NF1, BRAF, and NTRK3 fusions [1].

The discovery of mutations and the subsequent development of the first KIT tyrosine kinase inhibitor (TKI), imatinib (IM), in 2001, revolutionized the treatment of newly diagnosed locally advanced and metastatic GIST patients, who are commonly resistant to conventional chemotherapy [5]. Modular continuous flow synthesis of IM is reported to improve its yield [6]. The positive response of IM use with a median treatment duration of 55 months [7] is quite evident in some patients with specific molecular subtypes (KIT/PDGFRA wild type and PDGFRA D842V mutant); indeed, the PDGFRA D842V mutation creates a missense mutation conferring resistance to IM with poor prognostic outcomes in GIST patients [8]. The occurrence of secondary KIT mutations in more than 90% of GIST patients led to the approval of multi type II TKIs targeting KIT and PDGFRA [1], including sunitinib (SU) in 2006 and regorafenib (REG) in 2013 [9,10]. The TKI SU has a functional 3-methyleneoxindole group which gives it immunomodulation and angiogenesis inhibition; the median treatment duration in GIST patients is of 43 months as second-line therapy [11]. Among several published syntheses for REG, reaction of amine with the isocyanate ultimately delivered REG in 83% yield [12], with a median treatment duration of 7.6 months as third-line therapy in GIST patients [11]. However, the prevalence of KIT reactivation consequent to secondary resistance KIT mutations led to the approval of ripretinib (RIP) in 2021 [13], even though the synthesis of RIP was started by Flynn et al. in 2012 [14]. RIP inhibits the pocket at the juxtamembrane domain and activates the switch in the activation loop. Its broad targeting of primary and secondary mutations improves the progression-free survival (PFS) and overall survival (OS) of patients with a median duration of treatment of 3.7 months [1,15]. The PDGFRA D842V mutation, reported in 5% of all GISTs, together with its uncommon secondary KIT mutation (D816V) is intrinsically resistant to all TKIs. In a Korean study, the probability of stable disease was 25% after 24 weeks of treatment with SU [16]. Moreover, there is limited evidence relating mutational status to REG response in patients with the PDGFRA D842V mutation. The approval of avapritinib (AVA), a highly selective type I TKI in 2020 proved to be a milestone in PDGFRA D842V mutant GISTs with a median treatment duration of 23 months [17,18,19]. The original synthesis of AVA was reported by Houdous in 2017 and later improved by Chinese patent applications, reducing the synthesis to three steps using L-DBTA to increase the total yield and reduce the final production cost [14].

Considering their different mechanisms of action, including the development of secondary resistance mutations, TKIs differ in their effectiveness and safety profile [20] and in some adverse events (AEs) such as skin and gastrointestinal toxicity with IM [21]; anemia, hand foot syndrome, and hypertension with SU [22]; anemia, cognitive effects, and periorbital edema with AVA [23]; hand-foot skin reaction, hepatotoxicity, and hypertension with REG [24]; myalgia, palmar-plantar erythrodysesthesia syndrome (PPES), and weight loss with RIP [25].

Considering the recent approval of the last two TKIs and given their increased use in clinical practice, the spontaneous reporting system (SRS) database, the main tool in pharmacovigilance, plays a remarkable role in promptly characterizing the benefit/risk profile of these new drugs and consequently improving the quality of life of patients affected by GISTs. Currently, no original research on adverse drug reactions (ADRs) related to TKIs for the treatment of GISTs from the SRS database is available, and some questions remain to be answered, such as neurocognitive and psychiatric AEs. Premarketing studies have suggested a possible correlation between AVA and cognitive impairment [23,26], and the risk management plan of the European Medicines Agency (EMA) states that intracranial hemorrhage and cognitive effects are important identified risks for AVA [27]. Neuropsychiatric ADRs have a major impact on patients’ quality of life, especially those with GISTs. Some GIST patients may have a vulnerability to drug effects possibly driven by an immune-mediated process. The addition of AVA with increased penetration to the central nervous system may trigger a clinical cascade of neuropsychiatric deterioration [28]. However, no postmarketing data have evaluated neuropsychiatric AEs for TKIs. For all the above reasons, the aim of this study is to describe better ADRs and to evaluate the reporting frequency of some toxicities through the analysis of individual case safety reports (ICSRs) among TKIs approved for GISTs collected into the European SRS database with a focus on neuropsychiatric ADRs.

## 2. Materials and Methods

### 2.1. Data Sources and Selection Process

This is a retrospective, observational, pharmacovigilance study performed using the EudraVigilance (EV) database, a system designed for managing and analyzing information on suspected ADRs. The EV database is divided into two modules: the EV Clinical Trial module (EVCTM), for the collection of all ICSRs related to drugs that have not yet received marketing authorization and reported by sponsors, and the EV Post-Authorization Module (EVPM), which raises the ICSRs of suspected ADRs related to drugs authorized in the European Economic Area (EEA). Since 2012, the EMA has set up a publicly accessible online system for reporting suspected ADRs that are transmitted to EV electronically by the national medicine regulatory authorities and by marketing authorization holders (MAH). Several studies have been published that performed analyses using this SRS database [29,30,31,32].

All ADR ICSRs obtained from the EV database (www.adrreports.eu, accessed on 31 May 2022) using the line-listing function and recorded starting from the drug approval date up to 31 December 2021 with at least one of the following TKIs as suspected drugs were included: IM (from 1 June 2005 to 31 December 2021), SU (from 1 October 2006 to 31 December 2021), REG (from 1 January 2013 to 31 December 2021), AVA (from 1 October 2019 to 31 December 2021), and RIP (from 1 December 2020 to 31 December 2021). Before proceeding with the analysis, all premarketing ICSRs with supporting literature data were excluded. Moreover, to avoid therapeutic biases, all ICSRs having other indications and having more than one drug reported as suspected were also excluded.

All ICSRs that reported at least one neurological or psychiatric ADR were identified using the Medical Dictionary for Regulatory Activities (MedDRA^®^) System Organ Classes (SOCs) of “Nervous system disorders” or “Psychiatric disorders”. A detailed list of all High Level Terms (HLTs) and Preferred Terms (PTs) included in the analysis are reported in Supplementary Table S1.

### 2.2. Data Analyses

A descriptive analysis of the data obtained from EV was conducted to assess the demographic characteristics and variables related to the drugs under study. The analyses were carried out considering the demographic data of the patient (gender and age), information on the ADR (seriousness and outcome), primary source qualification, primary source country for regulatory purpose, and all available information on suspected drugs. The specific criteria regarding the seriousness of the ADRs (serious or not serious) were not available for all ICSRs, but ADRs were classified as serious if considered life-threatening or fatal, if due to hospitalization or the extension of an existing hospitalization, if due to permanent or significant disability/incapacity, if due to a congenital anomaly/congenital defect, or if classified as another important medical condition based on clinical judgment or the EV important medical event (IME) list, which is drafted by the EMA and updated every six months. The outcome of the ADRs was classified as “Recovered/Resolved”, “Recovering/Resolving”, “Recovered/Resolved with Sequelae”, “Not Recovered/Not Resolved”, “Fatal”, and “Unknown”. In the case of two or more ADRs with different results reported in a single ICSR, the result with the lowest resolution level was chosen for classification. ICSRs were classified as fatal if the outcome was “fatal”. The ADRs were analyzed according to MedDRA^®^ classification level by SOC, which are groupings by etiology and manifestation site, HLT based upon anatomy, pathology, physiology, etiology, or function, and PT considered a distinct descriptor for a symptom or sign. Moreover, the individual ADRs were sorted into the equivalent SOC and the PTs corresponding to the same clinical condition that were grouped under a unique term after a careful clinical evaluation.

A disproportionality analysis was conducted using the reporting odds ratio (ROR), with the corresponding 95% confidence interval (CI), to assess the frequency of reporting ADRs by MedDRA^®^ SOC for each TKI compared to all other TKIs with a focus on neuropsychiatric ADRs by HLT. The reference group includes all TKIs excluding the one of interest. The ROR and the 95% CI were calculated as a measure of disproportionality when the number of these cases was equal to or greater than five. Statistical analyses were conducted using the Statistical Package for the Social Science (SPSS) version 23.0 software for Windows (IBM Corp. SPSS Statistics). The time to onset (TTO) of neuropsychiatric ADRs was calculated only for ICSRs that reported both the duration of therapy and drug discontinuation as an action taken after the onset of the ADR. The duration of therapy, expressed in days, was retrieved from each ICSR if available. All neuropsychiatric ADRs with a higher frequency were carefully evaluated considering all PTs related to each HLT: ADRs were considered as expected for every TKI if acknowledged in the Summary of Product Characteristics (SmPCs) available at the time of the study by the EMA website.

## 3. Results

### 3.1. Characteristics of ICSRs

**Figure 1:**
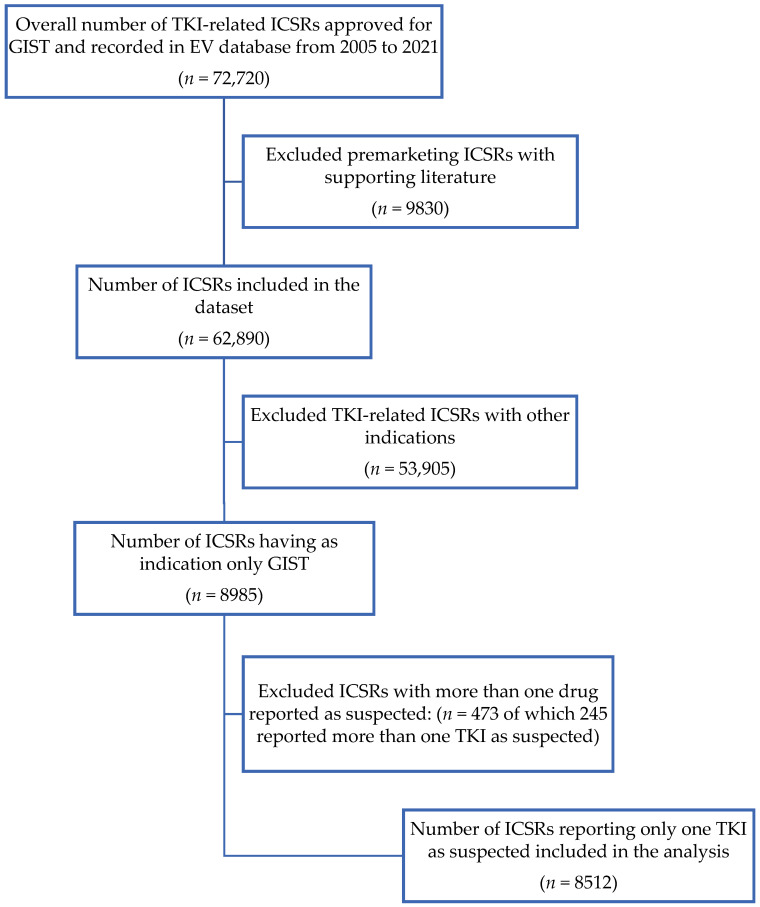
ICSR selection process. Abbreviations: EV = EudraVigilance; GIST = gastrointestinal stromal tumor; ICSRs = individual case safety reports; TKI = tyrosine kinase inhibitors.

A total of 72,720 TKI-related ICSRs approved for GISTs were collected in the EV database from 2005 to 2021. Of these, premarketing ICSRs with supporting literature (*n* = 9830) and TKI-related ICSRs with other indications (*n* = 53,905) were excluded. Furthermore, ICSRs with more than one drug reported as suspected (*n* = 473) were also excluded to avoid bias. The number of ICSRs involved in the analysis was 8512 reports (Figure 1): they were mainly related to IM (*n* = 4931; 57.9%), followed by SU (*n* = 2062; 24.2%) and AVA (*n* = 1112; 13.1%). A lower percentage of reports was associated with REG (2.7%) and RIP (2.1%).

**Figure 2:**
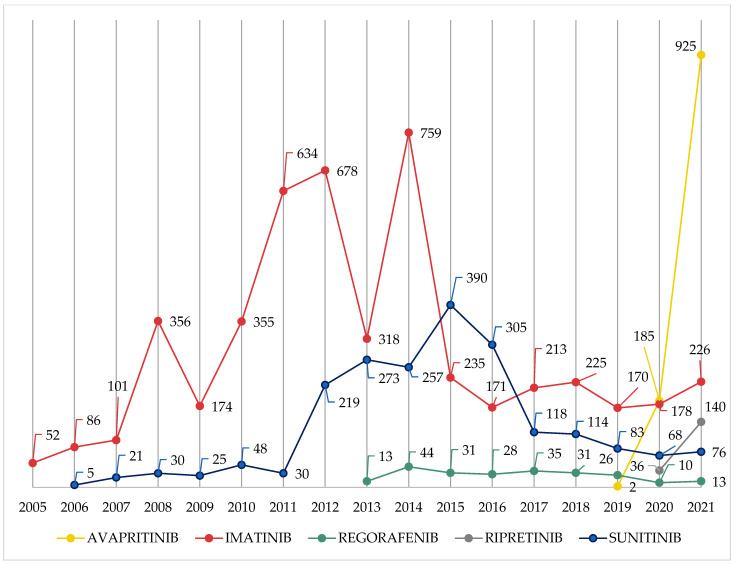
Trend over the years of TKI-related ICSRs with a GIST indication reported in EV database. Abbreviations: EV = EudraVigilance; GIST = gastrointestinal stromal tumor; ICSR = individual case safety report; TKI = tyrosine kinase inhibitors.

The trend over the years was controversial: AVA and RIP had an exponential increase, while IM and SU had a peak in 2014 and 2015, respectively (*n* = 759; 15.4% and *n* = 390; 18.9%, respectively); REG had a constant and low number of ICSRs (Figure 2).

Of the 8512 ICSRs, 4401 (51.7%) were related to male patients and 3802 (44.7%) to adults aged 18–64 years, except for AVA and RIP, which had higher percentages of elderly patients (55.8% and 50.0%, respectively). The majority of ICSRs were provided by healthcare professionals (*n* = 5718; 67.2%), mostly in countries belonging to non-European Economic Areas (non-EEA) (*n* = 7122; 83.7%). A total of 87.5% of all ADRs (*n* = 7450) were reported as serious, and approximately half of them were included in the “other medically important condition” serious criteria (*n* = 3557; 47.7%). However, AVA was associated with a lower number of serious ADRs (SADRs) (31.2%). Full resolution or recovering of ADRs was observed in 1043 (12.3%) and 810 (9.5%) ICSRs, respectively. However, ADRs were not yet resolved in 1704 patients (20.0%), while a quarter of total ICSRs (*n* = 2170; 25.5%) were fatal especially in SU-related reports (50.8%) (Table 1).

### 3.2. Characteristics of ADRs

The most reported ADRs were related to SOC general and administration site conditions (*n* = 3929; 46.2%), followed by neoplasm (*n* = 3051; 35.8%), gastrointestinal (*n* = 2195; 25.8%), skin (*n* = 1255; 14.7%), nervous (*n* = 1108; 13.0%), blood (*n* = 1035; 12.2%), and metabolism disorders (*n* = 995; 11.7%). A higher frequency of general and administration site conditions was reported for SU and RIP (*n* = 1343; 65.1% and *n* = 102; 58.0%, respectively), while neoplasms, intended as neoplasm progression or occurrence of metastases in other organs were mainly shown for IM and SU (*n* = 2042; 41.4% and *n* = 848; 41.1%, respectively). Gastrointestinal, nervous, and metabolism disorders were mostly reported for AVA (*n* = 404; 36.3%, *n* = 375; 33.7%, and *n* = 193; 17.4%), skin disorders for REG (*n* = 80; 34.6%), and blood disorders for SU and IM (*n* = 335; 16.2% and *n* = 611; 12.4%, respectively). The most frequently reported ADRs by HLT are listed in Table 2.

The probability of reporting ICSRs by SOC showed a higher probability of general and administration site conditions, especially asthenic conditions, with SU (ROR, 2.79; 95% CI 2.53–3.09), RIP (1.32; 1.18–2.23), and AVA (1.21; 1.06–1.37). Skin and subcutaneous tissue disorders had a higher probability of reporting for REG (3.20; 2.39–4.29), especially for the onset of PPES (*n* = 47) and RIP (1.50; 1.03–2.20), mainly for alopecia (*n* = 19). Cases belonging to musculoskeletal and connective tissue disorders, particularly arthralgia and musculoskeletal pain, had a higher probability for AVA (1.64; 1.33–2.04) and RIP (2.30; 1.49–3.54), while vascular disorders, especially hypertension, had a higher probability for SU (2.81; 2.36–3.35) and REG (1.70; 1.09–2.63). Considering cardiac disorders, higher reporting was noted for RIP (2.02; 1.23–3.31) and SU (1.42; 1.17–1.74), while hepatobiliary disorders were noted for REG (2.85; 1.93–4.22) and IM (1.28; 1.06–1.54).

Furthermore, AVA had a higher probability of eye disorders (3.55; 2.92–4.33), mainly characterized by lacrimation disorder and eye swelling (*n* = 61 and *n* = 58, respectively); immune system disorders (3.45; 2.23–5.35), especially seasonal allergy and hypersensitivity (*n* = 13 and *n* = 10, respectively); gastrointestinal disorders (1.79; 1.56–2.05), particularly diarrhea and nausea (*n* = 152 and *n* = 139, respectively); and metabolism disorders (1.73; 1.45–2.06), mainly decreased appetite (*n* = 75). Moreover, SU had a higher probability of endocrine (4.81; 3.24–7.13) disorders such as hypothyroidism (*n* = 38) and blood disorders (1.59; 1.38–1.83), including cases of thrombocytopenia and neutropenia (*n* = 169 and *n* = 130, respectively), IM of respiratory disorders (1.23; 1.08–1.44), mostly characterized by dyspnea and pleural effusion (*n* = 158 and *n* = 148, respectively); and RIP for infections and infestations (2.01; 1.28–3.17), especially urinary tract infection (*n* = 3) (see all RORs in Supplementary Table S2).

### 3.3. Neuropsychiatric ADRs

Neuropsychiatric ADRs were reported at least once in 1511 ICSRs (17.8%) for a total of 2162 ADRs (a mean of 1.4 neuropsychiatric ADRs for each ICSR). More than half of AVA-related reports had at least one neuropsychiatric ADR (*n* = 557; 50.1%). In contrast to all ICSRs, neuropsychiatric ICSRs were mainly related to elderly patients (*n* = 797; 52.7%) except IM; the gender difference remained (males 50.3% vs. females 46.5%). A higher percentage of neuropsychiatric ADRs were serious (*n* = 1097; 72.6%), but it was lower for AVA (*n* = 191; 34.3%), and approximately half of neuropsychiatric ADRs were not recovered/not resolved (*n* = 744; 49.2%), particularly for AVA (*n* = 428; 76.8%), while 9.1% were fatal, especially having SU as the suspected drug (*n* = 63; 17.6%) (Table 3).

**Figure 3:**
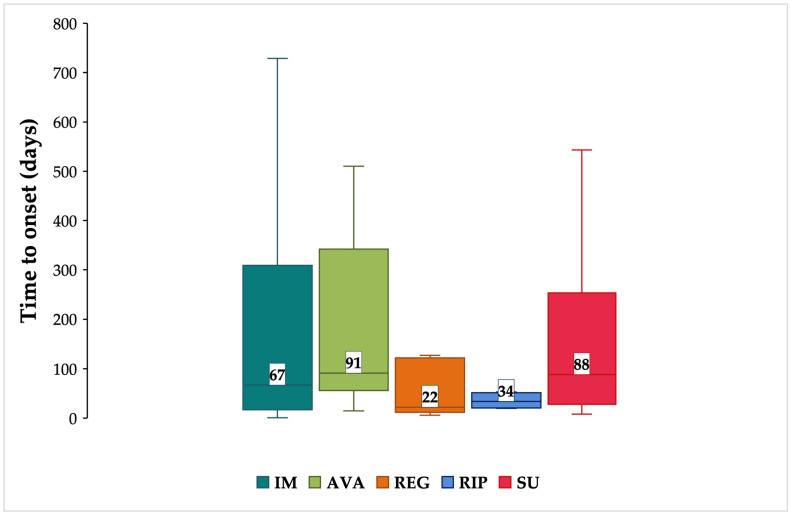
Time to onset of neuropsychiatric ADRs. Data are reported as box plots with the box drawn from Q1 to Q3 and the horizontal line drawn in the middle to denote the median. Abbreviations: AVA = avapritinib; IM = imatinib; REG = regorafenib; RIP = ripretinib; SU = sunitinib.

Only 124 ICSRs reported both duration of therapy and drug discontinuation as an action taken after the onset of the ADR. The median (Q1-Q3) TTO of neuropsychiatric ADRs was higher for AVA with 91 (56–342) days and SU with 88 (28.5–235) days (Figure 3).

Considering the probability of reporting neuropsychiatric ADRs, AVA was related to a higher reporting probability compared to all other TKIs: in detail, Psychiatric disorders (ROR 6.36; CI 95% 5.15–7.84) and Nervous system disorders (4.63; 3.99–5.37). Most neuropsychiatric ADRs by HLT with a higher probability of reporting when AVA was the suspected TKI were already known as common in the SmPC except for lumbar spinal cord and nerve root disorders intended as sciatica (ROR 4.46; CI 95% 1.58–12.54), olfactory nerve disorders, including anosmia and parosmia (8.02; 2.44–26.33), and hallucinations (excl sleep-related) (22.96; 8.45–62.36). For the other TKIs, some neuropsychiatric HLTs were associated with a higher probability of reporting: central nervous system hemorrhages and cerebrovascular accidents, especially cerebral infarction, were unknown for REG (3.19; 1.59–6.38); disturbances in consciousness not elsewhere classified (NEC), including somnolence, were not reported for RIP (3.15; 1.52–6.55); and SU neuropsychiatric ADRs by HLT were already reported in the SmPC with a different frequency (Table 4).

## 4. Discussion

This study could be considered the first to evaluate TKI-related ADR reports approved for the treatment of GISTs through the analysis of the European SRS database. The introduction of TKIs has radically changed the management of patients with GISTs. Although some TKIs, such as IM, have been used for many years, their use is not free from the onset of AEs. The distribution of ICSRs over the years has been certainly influenced by the different timing of approval and the clinical use of TKIs [5,9,18]: the higher incidence of IM-related ADRs is linked to its first approval; however, the exponential increase of AVA in the last year could be associated with its use as first-line treatment in metastatic GISTs with PDGFRA exon 18 mutation [2]. Looking at the gender and age distribution, ICSRs were mainly related to males and adult patients, which may be due to the higher prevalence of GISTs in male adult patients [2]. Conversely, IM- and SU-related serious AEs occurred more frequently in patients aged more than 70 years (14.7%) than in younger patients (3.8%) [33]. Additionally, most ADRs were serious except for AVA, probably due to the classification of GIST progression as IME, but this was controversial with what was observed in premarketing studies: SU showed a lower percentage of SADRs (approximately 20% of patients) [34]; this percentage increased in REG- and AVA-treated patients (61.4% and 65%, respectively) [35,36]. In the evaluation of seriousness, the reporting bias of SADRs due to regulatory authority obligations and to the priority of reporting serious rather than non-serious ADRs by healthcare professionals should be considered. Furthermore, 25.5% of ICSRs were fatal, but a causal relationship with the corresponding TKI cannot be sure, especially with most attention on ADRs resulting in death by SRS policies. Although a higher percentage of SU-related ADRs resulted in death than the other TKIs, the impact of the underlying GIST progression or the occurrence of metastases on the outcome of events cannot be excluded [37]. Moreover, the late approval of REG and RIP could create a bias in fatal cases due to the few ICSRs related to the use of third-line TKI therapy.

The analysis of ICSRs confirmed known TKI-related ADRs, which frequently involved general, gastrointestinal, skin, nervous, blood, and metabolism disorders. A higher frequency of musculoskeletal disorders was shown for RIP, which requires dose modifications when arthralgia or myalgia occur [13]. A greater frequency of cardiac disorders was already reported for RIP and SU, while hypertension was reported for SU and REG, both commonly observed with agents interfering with the vascular-endothelial growth factor (VEGF) pathways and its receptor VEGFR-2 [38,39]. Looking at hepatobiliary disorders, REG and IM appear to have a higher risk of increased hepatic enzymes and bilirubin levels [40,41]. In accordance with the disproportionality analyses, all ADRs with a higher frequency for AVA and SU have already been reported in their SmPC as very common [9,18]. In detail, the photosensitivity reactions with AVA may share a similar mechanism to the skin toxicities of other TKIs, such as IM, for the potent inhibition of the KIT gene [42]. Blood disorders, including neutropenia and thrombocytopenia, can be induced by SU through binding to Fms-like tyrosine kinase 3 (FLT-3) expressed on the surface of hematopoietic cells [43]. Moreover, long-term SU therapy could be associated with a risk of hypothyroidism, which can be managed by thyroid hormone replacement [34]. Considering IM, a higher frequency of respiratory disorders was shown possibly due to its kinase inhibition of PDGFRβ, which is expressed in pericytes and is involved in angiogenesis [44]. Conversely to the above already known ADRs, RIP had a higher frequency of infections, especially of the urinary tract: it is plausible that a decrease in white blood cell counts after RIP use could cause the patient to be at a higher risk of infections. Indeed, a phase 1 study showed 16 cases of urinary tract infections, mainly of grade 1–2, after the administration of RIP [45,46].

In addition to the above-described potential toxicities related to TKIs, evidence about neuropsychiatric ADRs deserves attention. A higher frequency of neuropsychiatric ADRs was reported for males and elderly patients with GISTs. A recent clinical study showed a higher incidence of cognitive effects in elderly patients [47], probably due to increased cognitive impairment and the dementia risk with increasing age [48]. Data on gender differences are still controversial: there seems to be a slightly higher incidence of neuropsychiatric effects in females [27] without any statistically significant difference between males and females [47]. Considering distribution by seriousness and outcome, a lower percentage of SADRs was noted for AVA, as confirmed in a previous clinical study that mainly reported grade 1/2 cognitive effects [47]. The increased reporting of unrecovered/unresolved AVA-related ADRs may be due to the median time to improvement of 16 weeks that leads to a missing follow-up of ICSRs [47]. The higher frequency of fatal ICSRs for SU was confirmed in the literature and could be related to the longer treatment duration, independent of tumor type [37]. The median TTO of neuropsychiatric ADRs was higher for AVA (approximately 13 weeks), but a slightly shorter median onset of cognitive effects (8.3 weeks) was highlighted in a previous study; this difference could be related to the cumulative dose of AVA and, therefore, to the different treatment duration [19].

The disproportionality analysis showed an increased risk of reporting a neuropsychiatric event with AVA compared to other TKIs used in GISTs. This could be due to the different mechanism of action of AVA compared to other TKIs considered type II inhibitors. Cognitive effects have generally not been reported for other TKIs except those crossing the blood–brain barrier, such as entrectinib or lorlatinib, despite not targeting PDGFRA [49]. However, AVA probably inhibits type 2 sodium channels, and allelic variants in the type 2 sodium channel have been associated with cognitive impairment [50]. Therefore, AVA-induced inhibition of both PDGFRA and the type 2 sodium channel may be involved in the development of neuropsychiatric ADRs. Moreover, considering the high selectivity of AVA, as a type I TKI, against the activation loop and the progression pattern of KIT-mutant GISTs, another hypothesis is the occurrence of secondary mutations in other regions of the kinase [1,51]. Indeed, in several premarketing studies, approximately 40% of AVA-treated patients had cognitive effects, including memory impairment, cognitive disorder, confusional state, and encephalopathy [35,47,52]. However, a possible correlation with pre-existing nervous or psychiatric comorbidities and the concomitant use of other drugs cannot be excluded. The use of TKIs with comedications is widely reported in the literature [53,54]; AVA is known to act on CYP3A, and co-administration with strong or moderate CYP3A inhibitors should be avoided because it may increase the plasma concentration, resulting in increased ADRs [53].

The analysis of AVA-related neuropsychiatric ADRs was consistent with information reported in SmPCs and the literature; however, lumbar spinal cord and nerve root disorders intended to be reported as sciatica were not reported in SmPCs and only 12 reports are available in the Food and Drug Administration Adverse Event Reporting System (FAERS) public dashboard [55]. In addition, evidence about olfactory disorders, such as parosmia and anosmia, is unknown. Smell disturbances were associated with other TKIs used in patients affected by GISTs: smell alterations (23%), reduced sense of smell (27%), and new unpleasant smells (20%) [56]. Furthermore, the onset of hallucinations, including visual and auditory ones, were unknown; however, the FDA multi-disciplinary review and evaluation for AVA suggests that the use of AVA could be associated with hallucinations occurring in 2.6% of patients with GISTs [57]. Another relevant ADR worthy of discussion was the higher frequency of cerebral infarction, clinically manifested as ischemic stroke, for REG; in a retrospective study, 4% of REG-treated patients had a cerebrovascular accident [11]. The neuroprotective role of VEGF is widely known, and inhibition of VEGFR by REG could be related to ischemic events [58]; indeed, the use of bevacizumab significantly increased the risk of cerebrovascular events [59]. A higher frequency of disturbances in consciousness, especially somnolence, was shown for RIP. Otherwise there is no evidence in the literature except for 16 cases reported in the FAERS public dashboard [55].

### Strengths and Limitations

This could be considered the first study focusing on ADRs, especially neuropsychiatric ones, related to the use of TKIs approved for GISTs and reported in the European SRS database. The SRS is a useful and cost-effective tool that aims to better characterize drug safety profiles through the collection of ICSRs; data from the European SRS database could generate potential safety signals of undetected ADRs from the premarketing phase, including rare and serious ones. Indeed, this pharmacovigilance method overcomes several premarketing study limitations such as the limited number of enrolled patients under controlled conditions and the short follow-up time [60,61]. The increasing use of TKIs in advanced GISTs and the recent approval of the last two TKIs, AVA and RIP, make further safety investigations on these drugs necessary, especially for neuropsychiatric ADRs. Patients with GISTs have worse health-related quality of life than the general population, especially when TKIs are used as later treatment lines with more negative effects also influenced by the duration of the tumor course [62]. For this reason, avoiding the onset of ADRs that could further worsen health-related quality of life and providing information on neuropsychiatric ADRs could be useful for oncologists in patient management as shown in previous studies [63,64].

Despite its strengths, the SRS database has some intrinsic limitations, such as the underreporting phenomena, the absence of denominators (i.e., the total number of GIST patients who have undergone treatment with TKIs), and the poor quality of information listed in each ICSR (i.e., seriousness and outcome, previous/current medical conditions, additional suspected or concomitant drugs) that could potentially affect the occurrence of analyzed ADRs [65]. In contrast to other SRS database studies, the use of the EV database does not allow the detection of all ICSR information, making it impossible to compute disproportionality analyses for all ICSRs related to SOC Nervous system disorders and Psychiatric disorders [42,66,67]. Therefore, further ad hoc studies are essential to establish the real neuropsychiatric safety profile of TKIs used in GISTs. Moreover, TKIs are prescribed in advanced and metastatic GISTs; consequently, it cannot be excluded that some serious and fatal ICSRs may be due to neoplasm progression and staging, to the onset of delayed ADRs, or to some comorbidities in GIST patients treated with TKIs [68]. However, even with its limitations, ICSRs obtained from the EV database help to better characterize TKI safety profiles, which is particularly important for preventing neuropsychiatric ADRs in GIST patients.

## 5. Conclusions

This study confirmed the fundamental role of the SRS database in evaluating ADRs related to TKIs approved for GISTs. The analyses of European ICSRs largely confirmed safety profiles described in each SmPC in terms of frequency, which frequently involved general, gastrointestinal, skin, nervous, blood, and metabolism disorders. However, AVA had an increased reporting probability of neuropsychiatric ADRs compared to all other TKIs. The computation of ROR highlighted some ADRs not reported in the literature and worthy of discussion: a higher frequency of nerve inflammation, olfactory dysfunction, and hallucinations was shown for AVA, a small increased reporting of cerebrovascular accident was noted for REG and disturbances in consciousness were noted for RIP. Neuropsychiatric ADRs have a strong impact on patients’ quality of life, especially those with GISTs. Considering that RIP and AVA have been recently available on the market, further studies are needed to increase the knowledge of the safety profiles of newly approved TKIs for GISTs and to better characterize neuropsychiatric disorders. Physicians should pay more attention to possible predictive factors of neuropsychiatric ADRs, including already existing nervous or psychiatric comorbidities. Therefore, further real-world studies and close collaboration among pharmacologists, neurologists, psychiatrists, and oncologists are helpful to identify new undetected ADRs early and to manage these effects appropriately in GIST patients.

## Figures and Tables

**Table 1:** Characteristics of TKI-related ICSRs approved for GIST collected into EV.

Characteristic, *n* (%)	AVA (*n* = 1112)	IM (*n* = 4931)	REG (*n* = 231)	RIP (*n* = 176)	SU (*n* = 2062)	Total (*n* = 8512)
Age Group						
Infant	1 (0.1)	2 (<0.1)				3 (<0.1)
Child	1 (0.1)	2 (<0.1)				3 (<0.1)
Adolescent		7 (0.1)			2 (0.1)	9 (0.1)
Adult	480 (43.2)	2027 (41.1)	111 (48.1)	85 (48.3)	1099 (53.3)	3802 (44.7)
Elderly	621 (55.8)	1783 (36.2)	82 (35.5)	88 (50.0)	785 (38.1)	3359 (39.5)
Not Specified	9 (0.8)	1110 (22.5)	38 (16.5)	3 (1.7)	176 (8.5)	1336 (15.7)
Patient Sex						
Female	511 (46.0)	1871 (37.9)	88 (38.1)	73 (41.5)	780 (37.8)	3323 (39.0)
Male	593 (53.3)	2373 (48.1)	121 (52.4)	102 (58.0)	1212 (58.8)	4401 (51.7)
Not Specified	8 (0.7)	687 (13.9)	22 (9.5)	1 (0.6)	70 (3.4)	788 (9.3)
Primary Source Qualification						
Healthcare Professional	91 (8.2)	4280 (86.8)	214 (92.6)	173 (98.3)	960 (46.6)	5718 (67.2)
Non-Healthcare Professional	1021 (91.8)	637 (12.9)	17 (7.4)	3 (1.7)	1102 (53.4)	2780 (32.7)
Not Specified		14 (0.3)				14 (0.2)
Primary Source Country for Regulatory Purposes						
European Economic Area	5 (0.4)	1082 (21.9)	83 (35.9)		220 (10.7)	1390 (16.3)
Non-European Economic Area	1107 (99.6)	3849 (78.1)	148 (64.1)	176 (100.0)	1842 (89.3)	7122 (83.7)
Serious	347 (31.2)	4719 (95.7)	196 (84.8)	176 (100.0)	2012 (97.6)	7450 (87.5)
Type of seriousness						
Caused/Prolonged Hospitalization	129 (37.2)	829 (17.6)	57 (29.1)	90 (51.1)	386 (19.2)	1491 (20.0)
Congenital Anomaly		1 (<0.1)				1 (<0.1)
Disabling	2 (0.6)	84 (1.8)	4 (2.0)		12 (0.6)	102 (1.4)
Life Threatening	2 (0.6)	88 (1.9)	7 (3.6)		32 (1.6)	129 (1.7)
Other Medically Important Condition	174 (50.1)	2694 (57.1)	101 (51.5)	54 (30.7)	534 (26.5)	3557 (47.7)
Results in Death	40 (11.5)	1023 (21.7)	27 (13.8)	32 (18.2)	1048 (52.1)	2170 (29.1)
Outcome						
Recovered/resolved	105 (9.4)	670 (13.6)	49 (21.2)	15 (8.5)	204 (9.9)	1043 (12.3)
Recovering/resolving	29 (2.6)	582 (11.8)	30 (13.0)	16 (9.1)	153 (7.4)	810 (9.5)
Recovered/resolved with sequelae		36 (0.7)	1 (0.4)		12 (0.6)	49 (0.6)
Not recovered/not resolved	674 (60.6)	697 (14.1)	48 (20.8)	14 (8.0)	271 (13.1)	1704 (20.0)
Fatal	40 (3.6)	1023 (20.7)	27 (11.7)	32 (18.2)	1048 (50.8)	2170 (25.5)
Unknown	264 (23.7)	1923 (39.0)	76 (32.9)	99 (56.3)	374 (18.1)	2736 (32.1)

AVA = avapritinib; EV = EudraVigilance; GIST = gastrointestinal stromal tumor; ICSR = individual case safety report; IM = imatinib; REG = regorafenib; RIP = ripretinib; SU = sunitinib; TKI = tyrosine kinase inhibitors.

**Table 2:** Distribution of main ADRs by MedDRA SOC and the top three HLTs within each SOC.

System Organ Class, *n* (%)	AVA (*n* = 1112)	IM (*n* = 4931)	REG (*n* = 231)	RIP (*n* = 176)	SU (*n* = 2062)	Total (*n* = 8512)
General disorders and administration site conditions	559 (50.3)	1847 (37.5)	78 (33.8)	102 (58.0)	1343 (65.1)	3929 (46.2)
General signs and symptoms NEC	157 (14.1)	292 (5.9)	7 (3.0)	31 (17.6)	786 (38.1)	1273 (15.0)
Asthenic conditions	282 (25.4)	350 (7.1)	37 (16.0)	33 (18.8)	237 (11.5)	939 (11.0)
Death and sudden death	32 (2.9)	481 (9.8)	7 (3.0)	24 (13.6)	321 (15.6)	865 (10.2)
Neoplasm benign, malignant and unspecified (incl cysts and polyps)	71 (6.4)	2042 (41.4)	57 (24.7)	33 (18.8)	848 (41.1)	3051 (35.8)
Gastrointestinal neoplasms malignant NEC		1077 (21.8)	46 (19.9)		682 (33.1)	1805 (21.2)
Neoplasms malignant site unspecified NEC	12 (1.1)	1482 (30.1)		6 (3.4)	14 (0.7)	1514 (17.8)
Neoplasms unspecified malignancy and site unspecified NEC	44 (4.0)	506 (10.3)		11 (6.3)	110 (5.3)	671 (7.9)
Gastrointestinal disorders	404 (36.3)	1139 (23.1)	42 (18.2)	46 (26.1)	564 (27.4)	2195 (25.8)
Nausea and vomiting symptoms	168 (15.1)	439 (8.9)	4 (1.7)	18 (10.2)	143 (6.9)	772 (9.1)
Diarrhoea (excl infective)	152 (13.7)	249 (5.0)	18 (7.8)	13 (7.4)	189 (9.2)	621 (7.3)
Gastrointestinal and abdominal pains (excl oral and throat)	60 (5.4)	234 (4.7)	6 (2.6)	14 (8.0)	79 (3.8)	393 (4.6)
Skin and subcutaneous tissue disorders	170 (15.3)	659 (13.4)	80 (34.6)	36 (20.5)	310 (15.0)	1255 (14.7)
Rashes, eruptions and exanthems NEC	55 (4.9)	342 (6.9)	22 (9.5)	9 (5.1)	46 (2.2)	474 (5.6)
Dermal and epidermal conditions NEC	41 (3.7)	127 (2.6)	7 (3.0)	5 (2.8)	117 (5.7)	297 (3.5)
Dermatitis ascribed to specific agent	4 (0.4)	44 (0.9)	51 (22.1)	7 (4.0)	116 (5.6)	222 (2.6)
Nervous system disorders	375 (33.7)	391 (7.9)	26 (11.3)	30 (17.0)	286 (13.9)	1108 (13.0)
Sensory abnormalities NEC	55 (4.9)	46 (0.9)	1 (0.4)		102 (4.5)	204 (2.4)
Neurological signs and symptoms NEC	75 (6.7)	72 (1.5)		8 (4.5)	44 (2.1)	199 (2.3)
Headaches NEC	51 (4.6)	46 (0.9)	1 (0.4)	6 (3.4)	45 (2.2)	149 (1.8)
Memory loss (excl dementia)	126 (11.3)	13 (0.3)			10 (0.5)	149 (1.8)
Blood and lymphatic system disorders	73 (6.6)	611 (12.4)	12 (5.2)	4 (2.3)	335 (16.2)	1035 (12.2)
Anaemias NEC	28 (2.5)	207 (4.2)	2 (0.9)	3 (1.7)	60 (2.9)	300 (3.5)
Thrombocytopenias	11 (1.0)	88 (1.8)	5 (2.2)	1 (0.6)	171 (8.3)	276 (3.2)
Leukopenias NEC	31 (2.8)	127 (2.6)	2 (0.9)		81 (3.9)	241 (2.8)
Metabolism and nutrition disorders	193 (17.4)	488 (9.9)	26 (11.3)	14 (8.0)	274 (13.3)	995 (11.7)
Appetite disorders	81 (4.3)	137 (2.8)	19 (8.2)	6 (3.4)	127 (6.2)	370 (4.3)
General nutritional disorders NEC	54 (3.3)	123 (2.5)	4 (1.7)	7 (4.0)	96 (4.7)	284 (3.3)
Metabolic disorders NEC	5 (1.3)	93 (1.9)			9 (0.4)	107 (1.3)

ADR = adverse drug reaction; AVA = avapritinib; excl = excluding; HLT = High Level Term; incl = including; IM = imatinib; MedDRA^®^ = Medical Dictionary for Regulatory Activities^®^; NEC = not elsewhere classified, which is a standard abbreviation used to denote groupings of miscellaneous terms that do not readily fit into other hierarchical classifications within a particular SOC; REG = regorafenib; RIP = ripretinib; SU = sunitinib.

**Table 3:** Characteristics of TKI-related neuropsychiatric ICSRs.

Characteristic, *n* (%)	AVA (*n* = 557)	IM (*n* = 528)	REG (*n* = 30)	RIP (*n* = 39)	SU (*n* = 357)	Total (*n* = 1511)
Age Group						
Adolescent		1 (1.2)			2 (0.6)	3 (0.2)
Adult	187 (33.6)	242 (45.8)	10 (33.3)	17 (43.6)	161 (45.1)	617 (40.8)
Elderly	365 (65.5)	220 (41.7)	14 (46.7)	21 (53.8)	177 (49.6)	797 (52.7)
Not Specified	5 (0.9)	65 (12.3)	6 (20.0)	1 (2.6)	17 (4.8)	94 (6.2)
Patient Sex						
Female	265 (47.6)	245 (46.4)	6 (20.0)	14 (35.9)	173 (48.5)	703 (46.5)
Male	287 (51.5)	247 (46.8)	23 (76.7)	25 (64.1)	178 (49.9)	760 (50.3)
Not Specified	5 (0.9)	36 (6.8)	1 (3.3)		6 (1.7)	48 (3.2)
Primary Source Qualification						
Healthcare Professional	33 (5.9)	398 (75.4)	28 (93.3)	39 (100)	217 (60.8)	715 (47.3)
Non-Healthcare Professional	524 (94.1)	127 (24.1)	2 (6.7)		140 (39.2)	793 (52.5)
Not Specified		3 (0.6)				3 (0.2)
Primary Source Country for Regulatory Purposes						
European Economic Area	5 (0.9)	159 (30.1)	14 (46.7)		40 (11.2)	218 (14.4)
Non-European Economic Area	552 (99.1)	369 (69.9)	16 (53.3)	39 (100)	317 (88.8)	1293 (85.6)
Serious	191 (34.3)	489 (92.6)	29 (96.7)	39 (100)	349 (97.8)	1097 (72.6)
Type of seriousness						
Caused/Prolonged Hospitalization	68 (35.6)	146 (29.9)	7 (24.1)	20 (51.3)	140 (40.1)	381 (34.7)
Disabling	2 (1.0)	25 (5.1)	1 (3.4)		3 (0.9)	31 (2.8)
Life Threatening	1 (0.5)	16 (3.3)	2 (6.9)		8 (2.3)	27 (2.5)
Other Medically Important Condition	117 (61.3)	238 (48.7)	13 (44.8)	17 (43.6)	135 (38.7)	520 (47.4)
Results in Death	3 (1.6)	64 (13.1)	6 (20.7)	2 (5.1)	63 (18.1)	138 (12.6)
Outcome						
Recovered/resolved		8 (1.5)	1 (3.3)		4 (1.1)	13 (0.9)
Recovering/resolving	14 (2.5)	80 (15.2)	1 (3.3)	6 (15.4)	45 (12.6)	146 (9.7)
Recovered/resolved with sequelae	24 (4.3)	69 (13.1)	10 (33.3)	3 (7.7)	38 (10.6)	144 (9.5)
Not recovered/not resolved	428 (76.8)	168 (31.8)	4 (13.3)	7 (17.9)	137 (38.4)	744 (49.2)
Fatal	3 (0.5)	64 (12.1)	6 (20.0)	2 (5.1)	63 (17.6)	138 (9.1)
Unknown	88 (15.8)	138 (26.1)	8 (26.7)	21 (53.8)	69 (19.3)	324 (21.4)

AVA = avapritinib; ICSR = individual case safety report; IM = imatinib; REG = regorafenib; RIP = ripretinib; SU = sunitinib; TKI = tyrosine kinase inhibitors.

**Table 4:** ROR of ICSRs with ADRs belonging to the SOC “Nervous system disorders” or “Psychiatric disorders” by High Level Term for the comparison of TKIs.

	AVA (*n* = 1112)			IM (*n* = 4931)		REG (*n* = 231)			RIP (*n* = 176)			SU (*n* = 2062)			Total (*n* = 1511)
	*n*	ROR (95% CI)	SmPC	*n*	ROR (95% CI)	*n*	ROR (95% CI)	SmPC	*n*	ROR (95% CI)	SmPC	*n*	ROR (95% CI)	SmPC	
Nervous system disorders															
Central nervous system hemorrhages and cerebrovascular accidents	2	NC		51	0.59 (0.41–0.86)	9	**3.19 (1.59–6.38)**	NR	4	NC		47	**2.26 (1.55–3.29)**	UN	113
Coordination and balance disturbances	33	**12.54 (7.04–22.35)**	C	6	0.10 (0.04–0.22)	0	-		0	-		12	0.96 (0.50–1.84)		51
Cortical dysfuntion NEC	33	**16.14 (8.61–30.25)**	C	6	0.11 (0.04–0.25)	0	-		0	-		8	0.64 (0.30–1.37)		47
Dementia (excl Alzheimer’s type)	13	**10.93 (4.52–26.43)**	C	6	0.29 (0.11–0.75)	0	-		0	-		2	NC		21
Disturbances in consciousness NEC	31	**2.07 (1.38–3.11)**	C	65	0.70 (0.50–0.99)	2	NC		8	**3.15 (1.52–6.55)**	NR	26	0.76 (0.50–1.18)		132
Encephalopathies NEC	2	NC		2	NC	4	NC		0	-		10	**3.92 (1.55–9.96)**	R	18
Headaches NEC	51	**3.58 (2.54–5.05)**	C	46	0.32 (0.22–0.45)	1	NC		6	2.02 (0.88–4.64)		45	1.36 (0.96–1.94)		149
Lumbar spinal cord and nerve root disorders	6	**4.46 (1.58–12.54)**	NR	7	0.63 (0.23–1.75	0	-		0	-		2	NC		15
Memory loss (excl dementia)	126	**40.99 (26.15–64.24)**	C	13	0.07 (0.04–0.12)	0	-		0	-		10	0.22 (0.12–0.42)		149
Mental impairment (excl dementia and memory loss)	72	**26.89 (16.16–44.77)**	C	12	0.11 (0.06–0.20)	2	NC		0	-		5	0.18 (0.07–0.44)		91
Narcolepsy and hypersomnia	6	**20.07 (4.05–99.55)**	C	0	-	0	-		0	-		2	NC		8
Nervous system disorders NEC	7	**11.71 (3.42–40.08)**	C	2	NC	1	NC		0	-		1	NC		11
Neurological signs and symptoms NEC	75	**4.24 (3.16–5.69)**	C	72	0.40 (0.30–0.54)	0	-		8	2.03 (0.98–4.19)		44	0.89 (0.63–1.24)		199
Olfactory nerve disorders	6	**8.02 (2.44–26.33)**	NR	5	0.60 (0.18–1.98)	0	-		0	-		0	0.00		11
Paraesthesias and dysaesthesias	24	**1.92 (1.22–3.04)**	C	43	0.48 (0.32–0.70)	2	NC		3	NC		36	**1.57 (1.05–2.36)**	C	108
Sensory abnormalities NEC	55	**2.53 (1.85–3.47)**	C	46	0.20 (0.15–0.28)	1	NC		0	-		102	**3.24 (2.45–4.28)**	VC	204
Speech and language abnormalities	26	**10.40 (5.62–19.22)**	C	9	0.19 (0.09–0.40)	3	NC		1	NC		4	NC		43
Transient cerebrovascular events	1	NC		5	0.28 (0.10–0.78)	2	NC		0	-		10	**3.92 (1.55–9.96)**	UN	18
Tremor (excl congenital)	7	**4.68 (1.78–12.32)**	C	7	0.51 (0.19–1.33)	0	NC		1	NC		2	NC		17
Psychiatric disorders															
Anxiety symptoms	40	**10.19 (6.23–16.67)**	C	16	0.23 (0.13–0.40)	2	NC		1	NC		8	0.42 (0.20–0.88)		67
Behavior and socialization disturbances	3	NC		3	NC	1	NC		0	-		0	-		7
Confusion and disorientation	39	**8.37 (5.22–13.42)**	C	17	0.23 (0.13–0.39)	1	NC		1	NC		13	0.70 (0.38–1.28)		71
Disturbances in initiating and maintaining sleep	65	**12.70 (8.41–19.18)**	C	23	0.21 (0.13–0.34)	1	NC		3	NC		9	0.30 (0.15–0.60)		101
Eating disorders NEC	3	NC		9	0.33 (0.15–0.72)	0	-		1	NC		16	**3.87 (1.86–8.06)**	VC	29
Emotional and mood disturbances NEC	7	**3.90 (1.53–9.93)**	C	9	0.65 (0.27–1.61)	0	-		0	-		3	NC		19
Hallucinations (excl sleep-related)	17	**22.96 (8.45–62.36)**	NR	4	NC	0	-		0	-		1	NC		22
Mood alterations with depressive symptoms	12	**6.72 (3.01–14.99)**	C	6	0.24 (0.10–0.61)	0	-		1	NC		5	0.82 (0.31–2.21)		24
Thinking disturbances	5	NC		0	-	0	.		0	-		2	NC		7

Significant RORs are in bold type. NC = not calculated because there were fewer than five reports or there were reports only for one drug; ADR = adverse drug reaction; AVA = avapritinib; C = common; CI = confidence interval; excl = excluding; HLT = High Level Term; ICSR = individual case safety report; IM = imatinib; NEC = not elsewhere classified, which is a standard abbreviation used to denote groupings of miscellaneous terms that do not readily fit into other hierarchical classifications within a particular HLT; NR = not reported; R = rare; REG = regorafenib; ROR = reporting odds ratio; RIP = ripretinib; SmPC = Summary of Product Characteristics; SU = sunitinib; SOC = System Organ Class; TKI = tyrosine kinase inhibitors; UN = uncommon; VC = very common.

## Data Availability

The datasets presented in this study can be found in online repositories. The names of the repository/repositories and accession number(s) can be found below: The datasets generated for this study will not be made publicly available. The European dataset in aggregated form is available online at www.adrreports.eu (accessed on 5 August 2022) while the access to the national, non-aggregated datasets require the approval of the National Authorities.

## Supplementary Materials

The following supporting information can be downloaded at: https://www.mdpi.com/article/10.3390/cancers15061851/s1, Table S1: System Organ Classes, High Level Terms, and corresponding Preferred Terms included in the analysis of neuropsychiatric ADRs. Table S2: ROR of ICSRs with ADRs by SOC.
