# Supplementary material for: Neuropsychiatric Adverse Drug Reactions with Tyrosine Kinase Inhibitors in Gastrointestinal Stromal Tumors: An Analysis from the European Spontaneous Adverse Event Reporting System
Source: Cancers (Basel). 2023 Mar 20;15(6):1851. doi: 10.3390/cancers15061851 (PMC10046586; doi:10.3390/cancers15061851)
Supplement: Supplementary file 1 [file cancers-15-01851-s001.zip › cancers-2271976-supplementary.pdf]

cancers

MDPI

Table S1. System Organ Classes, High Level Terms and corresponding Preferred Terms included in the analysis of neuropsychiatric ADRs.

System Organ Class	High Level Term	Preferred Term
Nervous system disorder	Abnormal reflexes	Areflexia
		Hyporeflexia
		Reflexes abnormal
	Acute polyneuropathies	Guillain-Barre syndrome
	Alzheimer's disease (incl subtypes)	Dementia Alzheimer's type
	Arterial infections and inflammations	Giant cell arteritis
	Central nervous system aneurysms and dissections	Carotid artery aneurysm
		Intracranial aneurysm
	Central nervous system haemorrhages and cerebrovascular accidents	Brain stem haemorrhage
		Cerebellar haemorrhage
		Cerebellar infarction
		Cerebral artery embolism
		Cerebral artery occlusion
		Cerebral haematoma
		Cerebral haemorrhage
		Cerebral infarction
		Cerebral ischaemia
		Cerebral thrombosis
		Cerebrovascular accident
		Haemorrhage intracranial
		Haemorrhagic stroke
		Ischaemic stroke
		Subarachnoid haemorrhage

		Thalamus haemorrhage
	Central nervous system vascular disorders NEC	Brain hypoxia
		Cerebral microangiopathy
	Cerebrovascular and spinal vascular disorders NEC	Migraine
	Coma states	Coma
		Coma hepatic
	Coordination and balance disturbances	Ataxia
		Balance disorder
		Coordination abnormal
		Dysstasia
		Nystagmus
	Cortical dysfunction NEC	Aphasia
		Apraxia
		Dysgraphia
		Visuospatial deficit
	Dementia (excl Alzheimer's type)	Dementia
		Dementia with Lewy bodies
	Demyelinating disorders NEC	Demyelination
	Disturbances in consciousness NEC	Altered state of consciousness
		Depressed level of consciousness
		Lethargy
		Loss of consciousness
		Somnolence
		Syncope
	Disturbances in sleep phase rhythm	Irregular sleep phase
		Irregular sleep wake rhythm disorder
	Dyskinesias and movement disorders NEC	Akathisia

		Akinesia
		Dyskinesia
		Hypokinesia
		Motor dysfunction
		Movement disorder
	Dyssomnias	Poor quality sleep
	Encephalopathies NEC	Encephalopathy
		Hypertensive encephalopathy
		Hypoxic-ischaemic encephalopathy
		Leukoencephalopathy
		Posterior reversible encephalopathy syndrome
	Encephalopathies toxic and metabolic	Hepatic encephalopathy
	Eye movement disorders	IIIrd nerve paralysis
		VIth nerve paralysis
	Facial cranial nerve disorders	Bell's palsy
		Facial nerve disorder
		Facial paralysis
		Facial paresis
	Generalised tonic-clonic seizures	Generalised tonic-clonic seizure
	Headaches NEC	Headache
	Hydrocephalic conditions	Hydrocephalus
	Increased intracranial pressure disorders	Brain oedema
		Idiopathic intracranial hypertension
		Intracranial pressure increased
	Lumbar spinal cord and nerve root disorders	Sciatica
	Memory loss (excl dementia)	Amnesia

		Memory impairment
		Transient global amnesia
	Mental impairment (excl dementia and memory loss)	Cognitive disorder
		Disturbance in attention
		Mental impairment
	Mononeuropathies	Carpal tunnel syndrome
		Nerve compression
	Motor neurone disease	Motor neurone disease
	Motor neurone diseases	Amyotrophic lateral sclerosis
	Multiple sclerosis acute and progressive	Multiple sclerosis
	Muscle tone abnormal	Hypertonia
	Muscular autoimmune disorders	Myasthenia gravis
		Myasthenic syndrome
	Narcolepsy and hypersomnia	Hypersomnia
	Nervous system disorders NEC	Central nervous system lesion
		Cerebral disorder
		Nervous system disorder
		Neurotoxicity
		Paraneoplastic neurological syndrome
	Neurologic visual problems NEC	Hemianopia homonymous
	Neurological signs and symptoms NEC	Dizziness
		Drooling
		Head discomfort
		Myoclonus
		Nervous system disorder
		Patient elopement

		Presyncope
		Unresponsive to stimuli
	Neuromuscular disorders NEC	Muscle spasticity
	Olfactory nerve disorders	Anosmia
		Parosmia
	Optic nerve disorders NEC	Optic neuritis
	Paraesthesias and dysaesthesias	Burning sensation
		Dysaesthesia
		Formication
		Hyperaesthesia
		Hypoaesthesia
		Paraesthesia
	Paralysis and paresis (excl cranial nerve)	Hemiparesis
		Hemiplegia
		Monoparesis
		Monoplegia
		Paralysis
		Paraparesis
		Paraplegia
		Paresis
		Peripheral paralysis
	Parkinson's disease and parkinsonism	On and off phenomenon
		Parkinsonism
		Parkinson's disease
	Peripheral neuropathies NEC	Axonal neuropathy
		Ischaemic neuropathy
		Neuritis

		Neuropathy peripheral
		Polyneuropathy
	Seizures and seizure disorders NEC	Convulsions local
		Epilepsy
		Seizure
	Sensory abnormalities NEC	Ageusia
		Complex regional pain syndrome
		Decreased vibratory sense
		Dysgeusia
		Hypogeusia
		Neuralgia
		Restless legs syndrome
		Sensory disturbance
		Sensory loss
		Taste disorder
		Vibratory sense increased
		Electric shock sensation
	Speech and language abnormalities	Dysarthria
		Incoherent
		Language disorder
		Repetitive speech
		Speech disorder
	Spinal cord and nerve root disorders NEC	Myelopathy
		Spinal cord compression
		Spinal cord disorder
	Structural brain disorders NEC	Brain injury
	Transient cerebrovascular events	Transient ischaemic attack

	Tremor (excl congenital)	Asterixis
		Tremor
	Vagus nerve disorders	Vocal cord paralysis
Psychiatric disorder	Abnormal behaviour NEC	Abnormal behaviour
		Staring
	Anxiety symptoms	Agitation
		Anxiety
		Nervousness
		Stress
		Tension
	Attention deficit and disruptive behaviour disorders	Attention deficit hyperactivity disorder
	Behaviour and socialisation disturbances	Aggression
		Paranoia
		Personality change
	Cognitive and attention disorders and disturbances NEC	Daydreaming
		Distractibility
		Executive dysfunction
	Confusion and disorientation	Confusional state
		Disorientation
	Deliria	Delirium
	Delusional symptoms	Delusion
	Depressive disorders	Depression
	Disturbances in initiating and maintaining sleep	Insomnia
	Eating disorders NEC	Anorexia nervosa
		Eating disorder
	Emotional and mood disturbances NEC	Emotional disorder

		Euphoric mood
		Frustration tolerance decreased
		Irritability
	Fear symptoms and phobic disorders (incl social phobia)	Claustrophobia
		Fear
		Fear of death
	Fluctuating mood symptoms	Mood swings
	Hallucinations (excl sleep-related)	Hallucination
	Impulse control disorders	Poriomania
	Increased physical activity levels	Restlessness
	Mental disorders NEC	Mental disorder
		Mental status changes
	Mood alterations with depressive symptoms	Decreased interest
		Depressed mood
		Psychomotor retardation
	Mood disorders NEC	Affective disorder
		Apathy
		Laziness
		Listless
	Panic attacks and disorders	Panic attack
	Parasomnias	Abnormal dreams
		Sleep talking
		Somnambulism
	Perception disturbances NEC	Near death experience
	Personality disorders NEC	Personality disorder
	Psychiatric symptoms NEC	Abulia
		Psychiatric decompensation

	Psychotic disorder NEC	Psychotic disorder
	Schizophrenia NEC	Schizophrenia
	Sexual desire disorders	Loss of libido
	Sleep disorders NEC	Sleep disorder
	Somatic symptom disorders	Illness anxiety disorder
	Stereotypies and automatisms	Bruxism
	Suicidal and self-injurious behaviour	Completed suicide
		Suicidal ideation
		Suicide attempt
	Thinking disturbances	Bradyphrenia
		Thinking abnormal
		Thought blocking

Table S2. ROR of ICSRs with ADRs by SOC.

System Organ Class	AVA (*n* = 1,112)		IM (*n* = 4,931)		REG (*n* = 231)		RIP (*n* = 176)		SU (*n* = 2,062)		Total (*n* = 8,512)
	n	ROR (95% CI)	*n*	ROR (95% CI)	*n*	ROR (95% CI)	*n*	ROR (95% CI)	*n*	ROR (95% CI)	
Genrl	559	**1.21 (1.06-1.37)**	1,847	0.43 (0.40-0.46)	78	0.59 (0.44-0.78)	102	**1.62 (1.18-2.23)**	1,343	**2.79 (2.52-3.09)**	3,929
Neopl	71	0.10 (0.08-0.13)	2,042	**1.80 (1.65-1.96)**	57	0.58 (0.42-0.79)	33	0.41 (0.28-0.60)	848	**1.35 (1.22-1.49)**	3,051
Gastr	404	**1.79 (1.56-2.05)**	1,139	0.72 (0.66-0.79)	42	0.63 (0.45-0.90)	46	1.02 (0.72-1.45)	564	1.11 (0.99-1.24)	2,195
Skin	170	1.05 (0.88-1.25)	659	0.77 (0.69-0.87)	80	**3.20 (2.39-4.29)**	36	**1.50 (1.03-2.20)**	310	1.03 (0.90-1.18)	1,255
Nerv	375	**4.63 (3.99-5.37)**	391	0.34 (0.30-0.39)	26	0.84 (0.55-1.29)	30	1.38 (0.92-2.08)	286	1.10 (0.95-1.27)	1,108
Blood	73	0.47 (0.37-0.60)	611	1.05 (0.93-1.20)	12	0.39 (0.22-0.70)	4	NC	335	**1.59 (1.38-1.83)**	1,035
Metab	193	**1.73 (1.45-2.06)**	488	0.67 (0.59-0.76)	26	0.96 (0.63-1.46)	14	0.65 (0.37-1.13)	274	1.22 (1.05-1.41)	995
Resp	108	0.99 (0.80-1.23)	522	**1.24 (1.08-1.44)**	17	0.73 (0.44-1.21)	18	1.05 (0.64-1.73)	168	0.77 (0.65-0.92)	833
Inv	81	0.90 (0.71-1.15)	433	**1.33 (1.13-1.56)**	10	0.52 (0.27-0.99)	19	1.42 (0.87-2.31)	132	0.74 (0.61-0.91)	675
Inj&P	231	**4.84 (4.05-5.80)**	204	0.34 (0.28-0.40)	20	1.23 (0.77-1.98)	43	**4.42 (3.07-6.37)**	113	0.69 (0.56-0.86)	611
Musc	117	**1.64 (1.33-2.04)**	314	0.75 (0.64-0.88)	18	1.10 (0.67-1.80)	26	**2.30 (1.49-3.54)**	136	0.89 (0.73-1.08)	611
Infec	76	0.96 (0.74-1.23)	348	0.99 (0.84-1.17)	16	0.98 (0.58-1.64)	23	**2.01 (1.28-3.17)**	140	0.94 (0.77-1.15)	603
Vasc	48	0.61 (0.45-0.83)	214	0.43 (0.36-0.51)	24	**1.70 (1.09-2.63)**	17	1.55 (0.93-2.60)	251	**2.81 (2.36-3.35)**	554
Eye	168	**3.55 (2.92-4.33)**	305	1.03 (0.86-1.23)	4	NC	2	NC	42	0.26 (0.19-0.36)	521
Card	23	0.31 (0.21-0.48)	283	0.99 (0.83-1.19)	14	1.06 (0.61-1.84)	19	**2.02 (1.23-3.31)**	151	**1.42 (1.17-1.74)**	490
Hepat	40	0.58 (0.41-0.80)	311	**1.28 (1.06-1.54)**	33	**2.85 (1.93-4.22)**	5	0.47 (0.19-1.16)	101	0.80 (0.64-1.00)	490
Renal	40	0.67 (0.48-0.94)	262	1.15 (0.95-1.41)	12	1.04 (0.57-1.88)	6	0.66 (0.29-1.51)	108	1.06 (0.85-1.32)	428
Psych	182	**6.36 (5.15-7.84)**	137	0.36 (0.29-0.44)	4	NC	9	1.09 (0.55-2.15)	71	0.66 (0.51-0.85)	403
Surg	53	**1.81 (1.33-2.47)**	136	0.85 (0.66-1.09)	3	NC	60	**21.94 (15.39-31.27)**	0	-	252
Endo	7	0.47 (0.22-1.02)	28	0.26 (0.17-0.40)	6	2.20 (0.95-5.10)	1	NC	63	**4.81 (3.24-7.13)**	105
Immun	31	**3.45 (2.23-5.35)**	43	0.63 (0.42-0.96)	1	NC	1	NC	16	0.66 (0.38-1.13)	92
Ear	15	1.62 (0.92-2.86)	45	1.02 (0.65-1.61)	1	NC	0	-	16	0.82 (0.47-1.42)	77
Repro	2	0.22 (0.05-0.89)	44	1.69 (0.98-2.89)	1	NC	1	NC	15	0.98 (0.55-1.75)	63

Cong	0	-	23	NC	0	-	0	-	1	NC	24
SocCi	7	**2.75 (1.14-6.65)**	6	0.24 (0.10-0.61)	2	NC	3	NC	6	1.04 (0.41-2.63)	24
Prod	5	2.57 (0.91-7.22)	12	1.45 (0.55-3.88)	0	-	0	-	1	NC	18
Preg	0	-	5	NC	0	-	0	-	0	-	5

Significant RORs are in bold type. NC, not calculated because there were fewer than five reports or there were reports only for one drug. ADR, adverse drug reactions; AVA, avapritinib; Blood, blood and lymphatic system disorders; Card, cardiac disorders; CI, confidence interval; Cong, congenital, familial and genetic disorders; Ear, ear and labyrinth disorders; Endo, Endocrine disorders; Eye, eye disorders; Gastr, gastrointestinal disorders; Genrl, general disorders and administration site conditions; Hepat, hepatobiliary disorders; ICSR, Individual Case Safety Report; IM, imatinib; Immun, immune system disorders; Infec, infections and infestations; Inj&P, injury, poisoning and procedural complications; Inv, investigations; Metab, metabolism and nutrition disorders; Musc, musculoskeletal and connective tissue disorders; Neopl, neoplasms benign, malignant and unspecified (incl cysts and polyps); Nerv, nervous system disorders; Preg, pregnancy, puerperium and perinatal conditions; Prod, product issues; Psych, psychiatric disorders; REG, regorafenib; Renal, renal and urinary disorders; ROR, reporting odds ratio; Repro, Reproductive system and breast disorders; Resp, respiratory, thoracic and mediastinal disorders; RIP, ripretinib; Skin, skin and subcutaneous tissue disorders; SocCi, social circumstances; SU, sunitinib; Surg, surgical and medical procedures; SOC, System Organ Class; Vasc, vascular disorders.
